# Comparison of Nasal Mucus Metabolites in Chronic Rhinosinusitis With and Without Polyps and Healthy Controls

**DOI:** 10.1002/oto2.70304

**Published:** 2026-09-13

**Authors:** Michael F. Armstrong, Kathleen R. Bartemes, Danielle E. Hunter, Marisa D. Griesel, Stephen G. Voss, Garret W. Choby, Carlos D. Pinheiro Neto, John B. Hagan, Rohit D. Divekar, Erin K. O'Brien

**Affiliations:** ^1^ Department of Otolaryngology–Head and Neck Surgery Virginia Commonwealth University Richmond Virginia USA; ^2^ Department of Otolaryngology–Head and Neck Surgery Mayo Clinic Rochester Minnesota USA; ^3^ Division of Allergic Diseases Mayo Clinic Rochester Minnesota USA

**Keywords:** biomarker, chronic rhinosinusitis, endoscopy, eosinophilic, inflammatory, metabolomics, nasal polyposis, neutrophilic, paranasal sinus, purulent

## Abstract

**Objective:**

To identify differences in nasal mucus metabolites from patients with chronic rhinosinusitis with nasal polyps (CRSwNP) and chronic rhinosinusitis without nasal polyps (CRSsNP) and healthy controls.

**Study Design:**

Cross‐sectional.

**Setting:**

Single institution tertiary care facility.

**Methods:**

Patients were assigned to groups based on the appearance of normal mucosa (control [n = 5]), diseased mucosa ([CRSsNP] [n = 6]), or polypoid mucosa ([CRSwNP] [n = 4]), respectively. Mucus was collected on swabs. Nasal mucus metabolites were quantified via untargeted metabolomics using liquid chromatography‐mass spectrometry (LC‐MS) for detection of all metabolites observable with the method. Metabolites in biochemical pathways implicated in sinonasal disease were further selected for analysis.

**Results:**

In the glycerophospholipid pathway, glycerophosphocholine and glycerol‐3‐phosphate were significantly elevated in controls (C) compared to the CRSwNP and CRSsNP groups (false discovery rate [FDR] = 0.03, C vs CRSwNP fold change [FC] = 3.83, C vs CRSsNP FC = 3.92 and FDR = 0.02, C vs CRSwNP FC = 3.05, C vs CRSsNP FC = 3.50, respectively). In the sphingolipid pathway, C24‐OH sulfatide and dihydroceramide were significantly elevated in CRSwNP compared to CRSsNP and control mucus (FDR = 0.01, CRSwNP vs C FC = 146.75, CRSsNP vs C FC = 45.55, CRSwNP vs CRSsNP FC = 3.22 and FDR = 0.05, CRSwNP vs C FC = 4.40, CRSwNP vs CRSsNP FC = 2.72, respectively).

**Conclusion:**

Glycerophosphocholine and sphingolipid nasal mucus metabolites differed between patients with subtypes of CRS and controls. Nasal mucus metabolites may aid in differentiating subtypes of sinusitis.

Chronic rhinosinusitis (CRS) is classified into 2 phenotypes: CRS with nasal polyps (CRSwNP) and CRS without nasal polyps (CRSsNP).^1^ CRSwNP is often characterized by type 2 inflammatory mediators including interleukin (IL)‐5 and immunoglobulin (Ig) E while CRSsNP is characterized by type 1 inflammatory mediators including interferon‐l and transforming growth factor‐β.^2^ However, recent work has shown overlapping inflammatory signatures for CRSsNP and CRSwNP limiting the utility of using endotypes and phenotypes to guide treatment choices in CRS.^3^ The dichotomy of CRSwNP versus CRSsNP was further challenged by a recent cluster analysis, which identified a mixed CRSwNP/CRSsNP group characterized by a moderate increase in IL‐5 concentration.^4^ This data highlights a need to identify biomarkers to reflect CRS pathophysiology and tailor therapy.

Metabolomics is an emerging technology that comprehensively measures metabolites and low‐molecular‐weight molecules in a biologic specimen.^5^ Two distinct methods are used in metabolomics: an untargeted approach, which measures all analytes in a sample including chemical unknowns, and a targeted approach, which measures predefined biologic compounds.^6^ There are 2 studies comparing the metabolomics of sinonasal tissue^7^ and serum^8^ from CRS patients and controls, but no studies analyzing metabolites in nasal mucus from CRS patients. This study aimed to identify metabolites of selected biochemical pathways associated with sinonasal disease using untargeted metabolomics data from the analysis of middle meatus mucus from patients with healthy nasal mucosa, patients with CRSwNP, and patients with CRSsNP.

## Methods

Patients presenting to a tertiary rhinology clinic were recruited for participation. No formal power calculation was performed because this was a pilot study. Ethical approval for this study was obtained from the Mayo Clinic Institutional Review Board #13‐004872 and all included subjects signed written consent. Exclusion criteria included (1) systemic steroid treatment in the last 4 weeks, (2) antibiotic treatment in the last 4 weeks, (3) history of biologic treatment for CRS, (4) history of cystic fibrosis or primary ciliary dyskinesia, and (5) age <18 or >75 years old. Rigid nasal endoscopy was performed on all patients by a fellowship‐trained rhinologist. Fifteen patients participated in the study. The healthy controls included 5 patients without polyps, purulence, or inflammatory mucus present on endoscopy. The CRSwNP group included 4 patients with inflammatory mucosa and polyps present in the middle meatus. The CRSsNP group included 6 patients with diseased mucosa and purulent mucus in the middle meatus (insert Table 1). Mucus was collected on swabs from the middle meatus in each patient, stored at −80°C, and processed in a single batch. Samples were extracted and prepared for liquid chromatography and mass spectrometry (LC‐MS).

**Table 1:** Participant Demographic and Clinical Data

Age (y)	Sex	Group	Endoscopy indication	Previous surgery	Asthma	Allergy	Immunodeficiency
							
36	M	Control	Debridement s/p surgery for silent sinus syndrome	Yes	No	No	No
56	F	Control	Septal deviation	No	No	No	No
61	M	Control	Surveillance s/p transsphenoidal pituitary approach	Yes	No	No	No
76	F	Control	Surveillance s/p inverted papilloma resection	Yes	Yes	No	No
58	F	Control	Surveillance s/p transsphenoidal pituitary approach	Yes	No	No	No
65	M	CRSsNP	CRSsNP	Yes	Yes	Yes	Yes, IgM
46	F	CRSsNP	CRSsNP	Yes	No	No	Yes, IgG2 and IgA
78	M	CRSsNP	CRSsNP	Yes	No	Yes	No
64	M	CRSsNP	CRSsNP s/p resection of infratemporal fossa acinic cell carcinoma with adjuvant radiotherapy	Yes	No	No	No
61	M	CRSsNP	CRSsNP s/p resection of maxillary sinus squamous cell carcinoma with adjuvant radiotherapy	Yes	No	No	No
73	F	CRSsNP	CRSsNP	Yes	No	No	No
61	M	CRSwNP	AERD and CRSwNP	Yes	Yes	Yes	No
52	M	CRSwNP	CRSwNP	No	Yes	Yes	No
78	M	CRSwNP	CRSwNP	Yes	Yes	Yes	No
49	F	CRSwNP	CRSwNP	Yes	No	Yes	No

An untargeted metabolomics approach was selected with the goal of identifying biomarkers that differentiate healthy from diseased nasal mucus. Metabolomic profiling was performed using liquid chromatography/time‐of‐flight mass spectrometry (6220 ToF MS; Agilent) operated in both positive and negative electrospray ionization modes, using a scan range of *m*/*z* 100 to 1200 at a resolution of 10,000 as described previously.^9^ Each mucus sample was analyzed in triplicate, and chromatographic separation was achieved using hydrophilic interaction liquid chromatography (hilic) and reverse phase (C18) liquid chromatography. Metabolite peak intensities and differential regulation of metabolites between groups were determined as described previously.^9^ Each metabolite was identified based on accurate molecular weight, mass‐to‐charge‐ratio (*m*/*z*), retention time, and comparison to library entries of purified known standards in the METLIN database (http://metlin.scripps.edu).

Metabolites in 3 pathways implicated in inflammatory disease (arachidonic acid, glycerophospholipid, and sphingolipid)^10^ and 3 pathways implicated in infectious disease (glycolysis, pyruvate, and pentose phosphate)^11^ were selected for analysis. Geometric means were calculated for the peak values of selected metabolites identified in each patient. Averaged peak values were analyzed using MetaboAnalyst 6.0 (http://www.metaboanalyst.ca/MetaboAnalyst/home.xhtml). Chi‐square test and analysis of variance (ANOVA) were used to compare categorical and continuous demographic data among groups with a *P* < .05 considered statistically significant. ANOVA with Fisher's LSD post hoc analysis was performed to compare differentially expressed metabolites among CRSwNP, CRSsNP, and healthy control mucus with a significant false discovery rate (FDR) defined as <0.05. Fold change (FC) analysis was performed to compare the absolute value of change between 2 group means with FC < 0.5 and FC > 2 considered meaningful.

## Results

There was a significant difference among groups regarding the presence of comorbid allergies with all CRSwNP patients having seasonal allergies compared to no control patients and one‐third of CRSsNP patients (*P* = .009). No significant differences were observed among groups regarding age, sex, previous surgery, asthma, and immunodeficiency (Table 2).

**Table 2:** Participant Demographic Data Comparison

	Control (n = 5)	CRSsNP (n = 6)	CRSwNP (n = 4)	*P*‐value
Age				.65
Mean (SD)	57.4 (14.3)	64.5 (11.0)	60 (13.0)	
Range	36, 76	46, 78	49, 78	
Sex, n (%)				.52
Male	2 (0.40)	4 (0.67)	3 (0.75)	
Female	3 (0.60)	2 (0.33)	1 (0.25)	
Previous surgery, n (%)				.45
Yes	4 (0.80)	6 (1.00)	3 (0.75)	
No	1 (0.20)	0 (0.00)	1 (0.25)	
Asthma, n (%)				.12
Yes	1 (0.20)	1 (0.17)	3 (0.75)	
No	4 (0.80)	5 (0.83)	1 (0.25)	
Allergy, n (%)				.009
Yes	0 (0.00)	2 (0.33)	4 (1.00)	
No	5 (1.00)	4 (0.67)	0 (0.00)	
Immunodeficiency, n (%)				.18
Yes	0 (0.00)	2 (0.33)	0 (0.00)	
No	5 (1.00)	4 (0.67)	4 (1.00)	

Metabolites from each of the 6 selected pathways were identified in each specimen; however, the identified metabolites were not present in every sample. In the inflammatory metabolic pathways, 14 arachidonic acid, 11 glycerophospholipid, and seven sphingolipid metabolites were detectable in nasal mucus. In the infectious metabolic pathways, 6 glycolysis, 5 pyruvate, and 3 pentose phosphate metabolites were detectable in nasal mucus (Table 3).

**Table 3:** Metabolites of Targeted Inflammatory and Infectious Pathways Identified in Nasal Mucus

Inflammatory pathways			Infectious pathways		
Arachidonic acid	Glycerophospholipid	Sphingolipid	Glycolysis	Pyruvate	Pentose phosphate
11, 12, 15‐THETA	1‐Acyl‐sn‐glycero‐3‐phosphocholine	Sphingosine‐1‐phosphate	Acetic acid	(R)‐Lactate	d‐Glucose
15‐HETE	Phosphatidylglycerol	Sulfatide	beta‐d‐Glucose	Acetic acid	Pyruvate
Arachidonic acid ethyl ester	Ethanolamine	Ceramide	d‐Glucose 6‐phosphate	Acetylenedicarboxylate	d‐Glycerate 2‐phosphate
Leukotriene B4	Glycerophosphocholine	Dihydroceramide	d‐Glycerate 2‐phosphate	Pyruvate	
Leukotriene E4	Glycerol 3‐phosphate	Sphingosine	Pyruvate	Succinate	
Phosphatidylcholine	Serine	Serine	Salicin 6‐phosphate		
Prostacyclin	Phosphatidylethanolamine	Lactosylceramide			
Prostaglandin D2	Phosphatidic acid				
Prostaglandin E2	Phosphodimethylethanolamine				
Prostaglandin F2alpha	sn‐glycero‐3‐Phosphoethanolamine				
Prostaglandin H2	Trolamine				
Prostaglandin I2					
Prostaglandin J2					
Thromboxane B2					

Abbreviations: HETE, hydroxyeicosatetraenoic acid; THETA, trihydroxyicosatrienoic acid.

**Figure 1:**
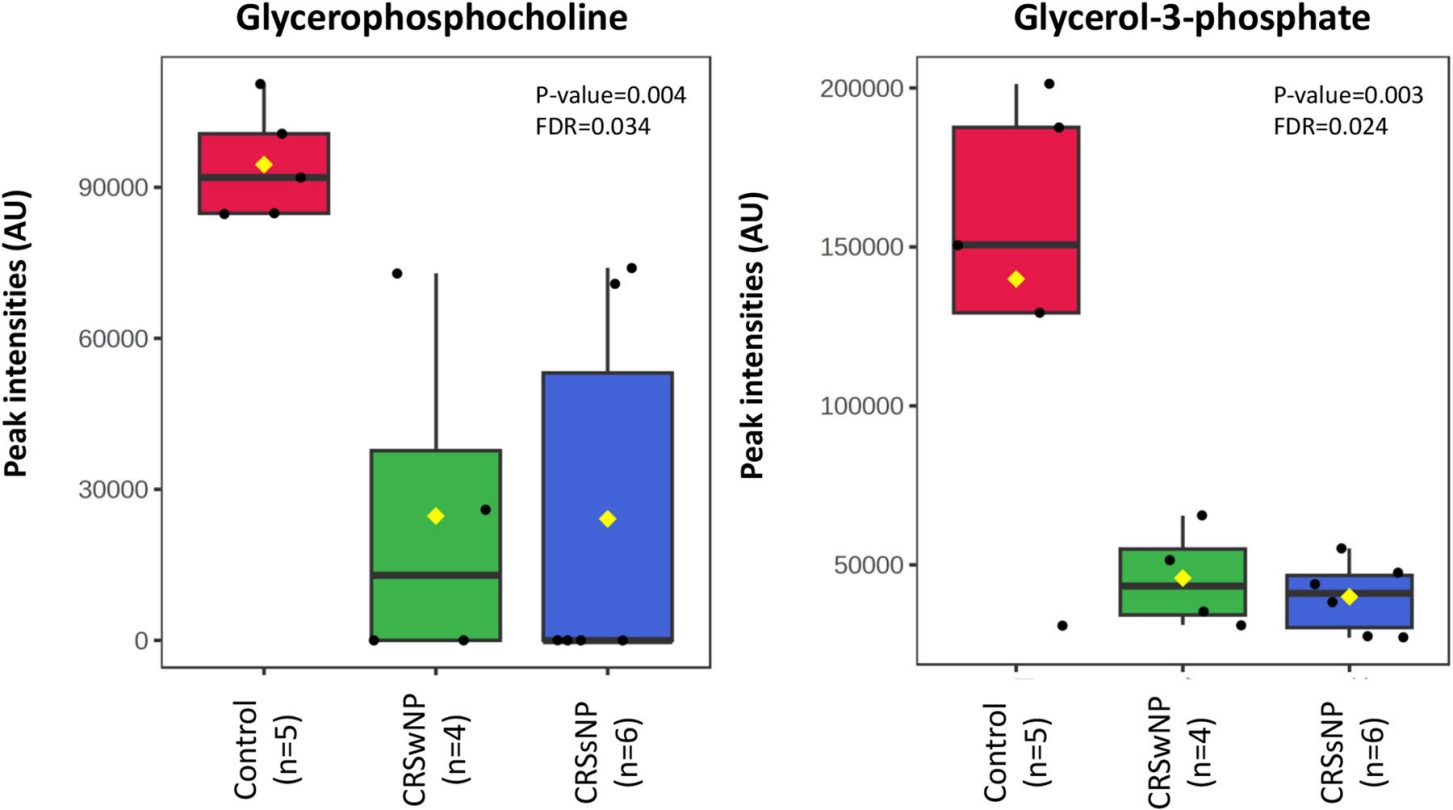
Glycerophosphocholine and glycerol‐3‐phosphate peak intensities after hydrophilic interaction and reverse phase liquid chromatography, respectively. Diamond = mean, line = median, box = Q1‐3 range, whisker = minimum and maximum, dot = sample peak intensity.

**Figure 2:**
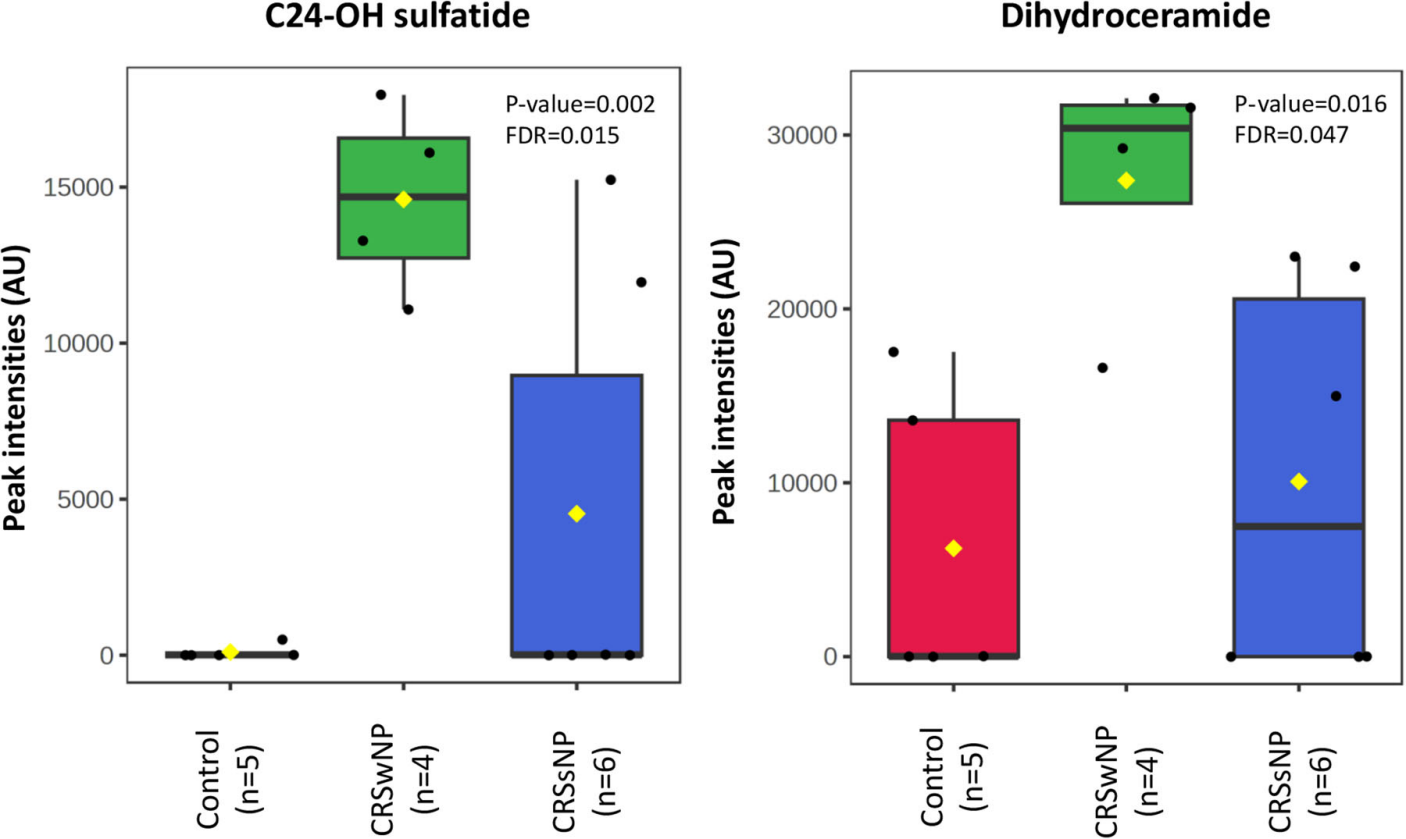
C24‐OH sulfatide and dihydroceramide peak intensities after hydrophilic interaction liquid chromatography. Diamond = mean, line = median, box = Q1‐3 range, whisker = minimum and maximum, dot = sample peak intensity.

Identified metabolites within each pathway were compared between the CRSwNP, CRSsNP, and control (C) groups. Significant differences were observed in glycerophospholipid and sphingolipid metabolite abundance. In the inflammatory glycerophospholipid pathway, glycerophosphocholine and glycerol‐3‐phosphate were significantly elevated in healthy controls compared to the CRSwNP and CRSsNP groups (FDR = 0.03, C vs CRSwNP FC = 3.83, C vs CRSsNP FC = 3.92 and FDR = 0.02, C vs CRSwNP FC = 3.05, C vs CRSsNP FC = 3.50, respectively, Figure 1). In the inflammatory sphingolipid pathway, C24‐OH sulfatide and dihydroceramide were significantly elevated in the CRSwNP group compared to the CRSsNP group and healthy controls (FDR = 0.01, CRSwNP vs C FC = 146.75, CRSsNP vs C FC = 45.55, CRSwNP vs CRSsNP FC = 3.22 and FDR = 0.05, CRSwNP vs C FC = 4.40, CRSwNP vs CRSsNP FC = 2.72, respectively, Figure 2).

## Discussion

Distinct metabolomic profiles of nasal mucus were observed among CRSwNP, CRSsNP, and healthy control patients. Glycerophosphocholine and glycerol‐3‐phosphate, both products of glycerophospholipid metabolism, were elevated in healthy control compared to CRS mucus. Glycerophospholipids are important components of the cell membrane structure and have previously been implicated in the pathogenesis of asthma.^12^ Similar to our study, a serum metabolomic study of eosinophilic asthmatics, non‐eosinophilic asthmatics, and healthy controls found a higher level of glycerophosphocholine in healthy controls, suggesting an anti‐inflammatory effect.^13^ Aberrant glycerophospholipid metabolism has also been implicated in CRS. An untargeted serum metabolomic study of patients with eosinophilic CRSwNP, those with noneosinophilic CRSwNP, and healthy controls found significantly elevated glycerophospholipid metabolite triethanolamine in the eosinophilic group compared to the noneosinophilic group.^8^ An untargeted tissue metabolomic study of patients with eosinophilic CRSwNP, noneosinophilic CRSwNP, CRSsNP, and healthy controls found significantly increased levels of glycerophosphocholine derivative 1‐palmitoyl‐2‐glutaryl‐sn‐glycero‐3‐phosphocholine in nasal mucosal tissue from patients with CRSsNP when compared to controls.^7^ Our findings of decreased glycerophosphocholine and its metabolite glycerol‐3‐phosphate in CRS mucus parallel the results from the asthma study. Taken together, this data suggests a role for phospholipid derivatives in the pathogenesis of CRS.

Sphingolipid metabolites C24‐OH sulfatide and dihydroceramide were significantly elevated in CRSwNP compared to CRSsNP and healthy control mucus. Sulfatides are multifunctional lipid molecules synthesized from ceramide that are presented on major histocompatibility complex class I‐like molecules and recognized by type 2 natural killer T cells (NKT).^14^ Their role in airway inflammation is unclear; one study demonstrated reduced proinflammatory cytokine secretion by mouse lung dendritic cells treated with sulfatide‐activated type II NKT cells in vivo, although this was not demonstrated in vitro.^15^ Elevated ceramide and its metabolic precursor dihydroceramide are thought to contribute to epithelial cell apoptosis and reactive oxygen species formation in mouse lung tissue after allergen challenge.^16^ Additionally, elevated lactosylceramide was observed in the serum of eosinophilic asthmatic patients.^13^ To the authors’ knowledge, this is the first report of sphingolipid metabolite differences identified among CRS patients and healthy controls. Our findings of increased ceramide concentrations in CRSwNP are consistent with the asthma studies and suggest a possible role in the pathogenesis of CRSwNP.

This study has several limitations. Because this is a pilot study, the associations found are still hypothetical and should be validated with larger sample sizes. Although this untargeted approach profiled all metabolites in nasal mucus samples, a targeted metabolomic approach may reduce the dominance of high‐abundance molecules and validate biomarkers implicated in sinonasal disease.^17^ While the control group was selected based on the absence of pathology at the time of nasal endoscopy, 4 of these patients had a history of previous surgery for inverted papilloma, silent sinus syndrome, or pituitary adenoma, which could potentially confound the results. Additional confounding factors include the variable presence of immunodeficiency, allergy, and asthma among the study participants. Finally, endoscopic appearance of the nasal mucosa was used to assign patients to the CRSwNP and CRSsNP groups. Repeat studies assigning patients to groups based on additional clinical parameters for subtyping disease is warranted.

## Conclusion

Using an untargeted metabolomics approach, differences in metabolites of selected biochemical pathways, specifically glycerophospholipid and sphingolipid metabolites, were identified in nasal mucus obtained from CRSwNP, CRSsNP, and healthy control patients. This pilot study showed nasal mucus metabolites could be utilized for identifying pathways for disease research. Further discovery of metabolites unique to CRS disease states may allow for the development of a nasal swab that can quickly identify and subtype CRS patients leading to targeted treatment.

## Author Contributions

**Michael F. Armstrong**, MD, study design, data collection and analysis, manuscript author and final approval; **Kathleen R. Bartemes**, PhD, study design, data analysis, manuscript final approval; **Danielle E. Hunter**, data collection, manuscript final approval; **Marisa D. Griesel**, data collection, manuscript final approval; **Stephen G. Voss**, data collection, manuscript final approval; **Garret W. Choby**, MD, data collection, manuscript final approval; **Carlos D. Pinheiro Neto**, MD, PhD, data collection, manuscript final approval; **John B. Hagan**, MD, manuscript final approval; **Rohit D. Divekar**, MBBS, PhD, manuscript final approval; **Erin K. O'Brien**, MD, study design, data collection and analysis, manuscript final approval.

## Disclosures

### Competing interests

None.

### Funding source

Funding for this study was provided by the Mayo Clinic Department of Otolaryngology, Rochester, MN.
